# Hospitalization- and death-related financial and employment effects in parents of children with life-limiting conditions: a fixed-effects analysis

**DOI:** 10.1007/s00431-024-05680-7

**Published:** 2024-07-10

**Authors:** Stefan Mitterer, Karin Zimmermann, Günther Fink, Michael Simon, Anne-Kathrin Gerber, Eva Bergsträsser

**Affiliations:** 1 Institute of Nursing Science, Department of Public Health, University of Basel, Bernoullistrasse 28, CH-4065 Basel, Switzerland; 2 Paediatric Palliative Care and Children’s Research Center, University Children’s Hospital Zurich, Zurich, Switzerland; 3 Swiss Tropical and Public Health Institute, University of Basel, Basel, Switzerland

**Keywords:** Child, Financial, Cost, Parent, Family, Life-limiting

## Abstract

The purpose of this study is to investigate out-of-pocket non-medical expenses and employment-related outcomes in families of children with life-limiting conditions, specifically, to quantify the financial and employment implications of two events: a child’s hospitalization and death. This cohort study used panel data collected prospectively for a larger study investigating the effectiveness of specialized pediatric palliative care. Participants were recruited by medical professionals between November 2019 and May 2022 at four Swiss children’s hospitals. The care follow-up and bereavement follow-up assessments were 330 and 300 days, respectively. We measured out-of-pocket non-medical expenses, individual full-time equivalent units, and personal income, as well as sick leave and vacation days taken. Analyses included descriptive statistics and two-way linear fixed-effects regressions. The analysis included 110 parents (mothers *n* = 59, fathers *n* = 51) of 61 children. Children were hospitalized for a median of 7 days (interquartile range 0–21, range 0–227). The fixed-effects models found a positive association between hospitalization, i.e., length of stay, and travel and accommodation expenses (coefficient 4.18, 95% confidence interval 2.20–6.16). On average, for each week of hospitalization, parents spent an additional 29 Swiss francs on travel and accommodation. During the 300-day bereavement follow-up, six (26%) of 23 parents increased their work commitments, while one reported a decrease.

*Conclusions*: Families incur higher travel and accommodation expenses during hospitalization than during non-hospitalized periods. Instrumental support, e.g., parking vouchers, can help families minimize these costs. Future studies should investigate whether early return to work during bereavement is driven by economic considerations or a desire for distraction.

*Clinical trial registration*: Data analyzed in this study were collected as part of a clinical trial, registered on ClinicalTrials.gov, No. NCT04236180, 15 March 2019

**Table:** 

**What Is Known:** *• Families of children with life-limiting conditions are at risk of substantial financial burden from high out-of-pocket medical expenses.* *• It is also known that parents often have to incur out-of-pocket non-medical expenses and reduce their work commitments. Little is known about the economic consequences of losing a child to a life-limiting condition.* **What Is New:** *• We provide new longitudinal evidence on the hospitalization- and death-related financial and employment implications for families of children with life-limiting conditions.* *• Child hospitalizations add to families’ financial burden through increased travel and accommodation expenses. Work commitments rose during early bereavement.*

**Supplementary Information:**

The online version contains supplementary material available at 10.1007/s00431-024-05680-7.

## Introduction

Parenting a child with a life-limiting condition (LLC), i.e., conditions “for which there is no reasonable hope of cure and from which children will die” [1, p.10], has an enormous impact on the life of these children’s parents, adversely affecting their physical, psychological, emotional, and social health [2–6]. In addition, these parents are likely to experience substantial economic adversity [7]. Previous research suggests that financial burden is driven by high out-of-pocket (OOP) medical spending, i.e., deductibles and co-payments [8–10]. However, the costs of taking care of a child with a LLC go beyond medical costs: parents often face OOP non-medical expenses and employment-related income losses [11–13]. For instance, Canadian studies revealed that following a child’s cancer diagnosis, 64% of mothers and 16% of fathers left their jobs [14] and that illness-related travel and food expenses accounted for nearly three quarters of families’ OOP non-medical expenses [15].

Over the course of a LLC, parents have to navigate a range of challenging and traumatic events, including unstable phases of illness, hospital admissions, and even their child’s death, any of which can increase their psychological, emotional, social, and financial burdens [2–6, 16–19]. For example, families may face extra expenses for traveling to and staying overnight at the hospital. Repeated and lengthy hospitalizations are common for children with LLCs [20, 21]. However, few studies have examined the effects of a child’s hospitalization on OOP non-medical expenses and parental employment [9, 22–26]. None of these studies is both LLC-specific and longitudinal [9, 22–26]. Having LLC-specific evidence is important since the economic impact is likely accentuated in this population because of higher healthcare resource utilization (e.g., repeated and lengthy hospitalizations) and the manifestation of financial burden over time.

The same is true for evidence on the financial and employment implications of a child’s death. Losing a child is a traumatic, life-changing event [27–31]. Conducting research during early bereavement is challenging, as parents are navigating an unimaginably difficult period of their lives [32]. As a result, studies about the economic consequences of grief are largely absent from the literature. Nevertheless, the financial and employment implications of losing a child to a LLC can be considerable [33].

For this study, we investigated the financial and employment implications of both hospitalization and bereavement. More specifically, the study’s first aim was to explore how a child’s hospitalization influences the families’ OOP non-medical expenses as well as the parents’ income and employment over a 330-day period. Its second aim was to assess changes in parental income and employment over the first 300 days of bereavement.

## Methods and materials

This cohort study is a secondary analysis of a prospectively collected panel dataset. The dataset was collected through the “Specialised Paediatric PAlliativE CaRe: Assessing family, healthcare professional and health system outcomes in a multi-site context of various care settings (SPhAERA)” study, which aimed to evaluate the effectiveness of a Swiss specialized pediatric palliative care (SPPC) program [34]. The study was conducted between November 2019 and May 2023 with the approval of the responsible Swiss ethics committees (BASEC-Nr. 2019–01170) and is registered in ClinicalTrials.gov (NCT04236180). Further information regarding the SPhAERA study can be found elsewhere [34].

### Participants and recruitment

This study’s population consisted of children with a LLC and their parents (mothers and/or fathers). To be included for study participation, children had to be 0–18 years of age and their families had to be proficient in either German or French. Children whose life expectancy was < 48 h and neonates with medical complications due to prematurity and/or birth complications treated in a neonatal intensive care unit were excluded. Children subject to child protection regulations and their parents were ineligible for study participation.

Participants were recruited between November 2019 and May 2022 at four Swiss study centers, i.e., four (university) children’s hospitals. Potential participants were screened for eligibility by the leading medical professional in collaboration with the study team. In one study center, participants were recruited consecutively when they entered the local SPPC program. In the other three study centers, participants potentially in need of SPPC were convenience recruited by the responsible medical professional. Parents provided written informed consent. All families in the study had mandatory health insurance, largely shielding them from OOP medical expenses.

### Data collection and variables

#### Data collection

The study duration was 330 days including nine assessment timepoints. In the event of a child’s death during this period, parents remained enrolled for an additional 300 days (two assessments), starting from the date of death. Diagnostic information and healthcare resource utilization data were extracted from routine data via chart reviews at baseline (day 0) and at eight care follow-up assessments. Healthcare resource utilization data collected at days 15 and 30 were merged to be in line with the collection of economic data, which started at day 30. See Table 1 for a detailed overview of assessment timepoints and variables. Parent and family characteristics and economic data were collected via pseudonymized paper-pencil self-report questionnaires, either distributed in hospital or sent via mail to families’ homes.

**Table 1:** Overview of assessment timepoints and variables

Study periods	Care follow-up assessment									Bereavement follow-up assessment	
Assessment timepoints, in days	0^a^	15	30	60	90	120	150	240	330	120^b^	300^b^
Variables											
Parent, family, and child characteristics	x										
Exposure											
Hospital length of stay		x	x	x	x	x	x	x	x		
Outcome											
Out-of-pocket expenses											
Home health-care supplies			x	x	x	x	x	x	x		
Travel and accommodation			x	x	x	x	x	x	x		
Childcare and home help			x	x	x	x	x	x	x		
Special and extraordinary			x	x	x	x	x	x	x		
Employment and income											
Full-time equivalent unit			x	x	x	x	x	x	x	x	x
Income			x	x	x	x	x	x	x	x	x
Work absenteeism											
Sick leave			x	x	x	x	x	x	x	x	x
Vacation			x	x	x	x	x	x	x	x	x
Other variables											
Financial support			x	x	x	x	x	x	x		

^a^Baseline assessment

^b^Days after the child’s date of death

#### Exposure variable

To explore associations between hospitalization and family economic outcomes, we used child hospital length of stay (LoS, days) as our exposure measure. For each assessment period, the number of days a child was hospitalized in one of the participating (university) children’s hospitals was recorded.

#### Outcome variables

In addition to a range of OOP non-medical expenses, outcome variables included individual full-time equivalent (FTE) units, personal income, and work absences, i.e., sick leave and vacation days (Table 1). While work absenteeism may not have immediate financial implications, it potentially hampers parents’ long-term career perspectives [35]. Families’ OOP non-medical expenses included illness-related expenses for home healthcare supplies, travel and accommodation, childcare and home help, and special and extraordinary purchases, e.g., home modifications. To measure work commitment and income loss, each parent’s FTE unit and income were recorded at study start. At each care follow-up assessment, parents who had experienced work and/or income-related changes were asked to provide their new FTE unit and/or income. Work absences (both paid and unpaid) of employed parents included two variables: sick leave days and vacation days. In the bereavement follow-up, individual FTE units, income, and work absenteeism, including compassionate days, were measured. OOP non-medical expenses and income data were collected using ordinal categories. All other variables were collected using free text fields. All expenses and income were measured in Swiss francs (CHF).

#### Other variables

We collected data on a number of relevant diagnostic and socio-demographic/economic characteristics (Table 2). We also assessed the number of hospitalizations and the amount of financial support families received, including support from all sources such as government, charities, and relatives.

**Table 2:** Parent, family, and child characteristics

Parents	(*n* = 110)^a^	Families	(*n*** = **61)^a^
Parents, *n* (%)		Household income in CHF^c^, *n* (%)	
Mothers/fathers	59 (54%)/51 (46%)	< 100,000	25 (41%)
Age in years		100,000–200,000	26 (43%)
Mean (SD)	38.7 (6.5)	> 200,000	8 (13%)
Nationality, *n* (%)		Missing	2 (3%)
Switzerland	85 (77%)	Home-hospital travel distance, *n* (%)	
Other	25 (23%)	0–20 km	23 (38%)
Time others have lived in Switzerland, *n* (%)		21–50 km	27 (44%)
> 10 years	18 (72%)	> 50 km	11 (18%)
< 10 years	7 (28%)		
Marital status, *n* (%)		Children	(***n*** = 61)
Married, partnership	100 (91%)	Gender, *n* (%)	
Other	10 (9%)	Female/male	34 (56%)/27 (44%)
Living situation, *n* (%)		Age in years	
Couple family with child(ren)	96 (87%)	Median (IQR)	3.6 (0.7–8.6)
Other	14 (13%)	Range	0.0–15.5
Number of children^b^		Diagnosis, *n* (%)	
Median (IQR)	2.0 (1.0–2.0)	Neurological	41 (67%)
Range	1.0–5.0	Cardiological	10 (16%)
Missing,* n* (%)	1 (1%)	Oncological	6 (10%)
Education, *n* (%)		Other	4 (7%)
Primary/secondary education or high school	8 (7%)	Illness duration in days^d^	
Vocational training	38 (35%)	Median (IQR)	357.0 (44.0–1827.0)
College of higher education	36 (33%)	Range	4.0–5640.0
University degree	28 (25%)	Place of care at study entry, *n* (%)	
Occupational status, *n* (%)		Hospital	24 (39%)
In employment	95 (86%)	Home	33 (54%)
Not in employment	15 (14%)	Other	4 (7%)

*SD* standard deviation, *IQR* inter quartile range, *CHF* Swiss francs

^a^A family (two parents) of twins was counted twice because both children had an LLC and participated in the study

^b^Number of children includes the child with an LLC

^c^Gross annual household income, Swiss average lies at CHF157,008[48]

^d^Illness duration gives the number of days between the date of diagnosis of the LLC and the date of study entry

### Statistical analyses

Descriptive statistics were used to provide an overview of parent, family, and child characteristics, to report on the financial support families received and to describe financial and employment implications. Missing data were analyzed using Little’s test [36].

To enhance our understanding of how changes in hospitalization (e.g., spending an additional day in hospital) influences families’ economic outcomes, we used two-way linear fixed-effects models [37, 38]. Our hypothesis was that periods with hospitalization are more costly for families due to increased cost (e.g., travel and transport), but may also be lower due to lower need for other purchases (e.g., home healthcare supplies). The empirical model is specified in Supplemental Material 1, where additional explanations are also provided.

All analyses were performed using the R statistical software (version 4.1.2) [39]. The regression analyses were performed using the R plm package (version 2.6-2) [40]. A *p*-value of < 0.05 was applied.

#### Robustness check

We checked the robustness of our findings via random-effects, complete-case, and subgroup analyses. Fixed- and random-effects models were compared using the Hausman test (Supplemental Table 1) [41]. Additional details regarding the robustness check can be found in Supplementary Material 1.

## Results

### Study participants

**Fig. 1:**
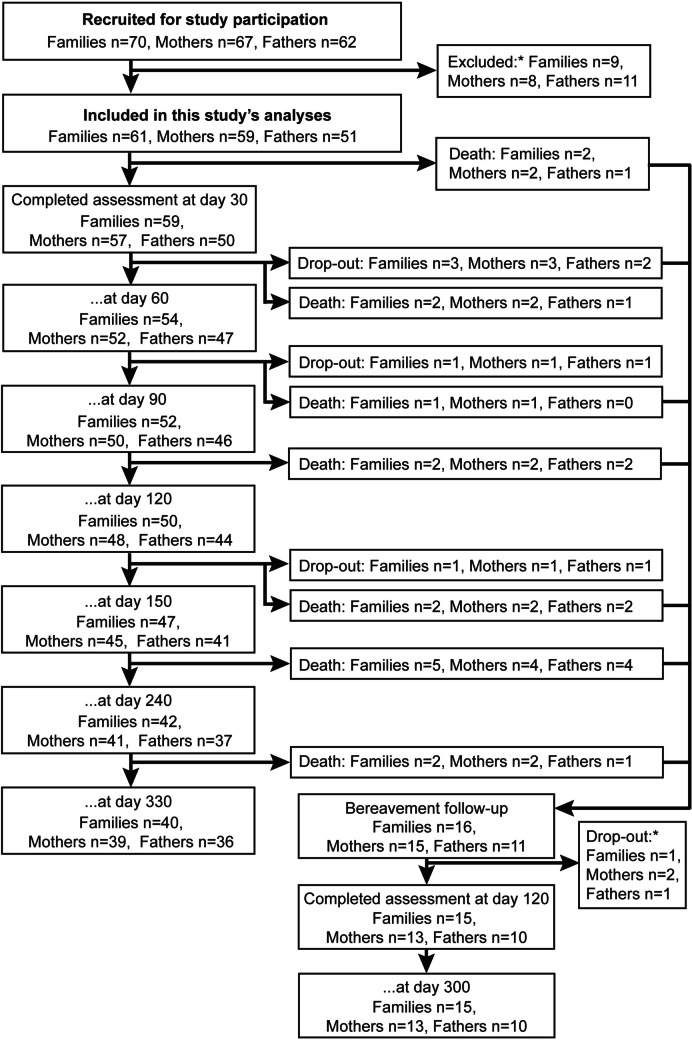
Flow diagram of study participants. The number of families is equal to the number of children; one family of twins was counted twice. Parents/families who did not complete assessments at day 30 or participate in the bereavement follow-up were excluded. *Only one parent (mother or father) was excluded/dropped-out

The inclusion criteria were met by 160 of 280 screened families. Of these 160 families, 70 consented for study participation. A family of twins was counted twice because both children participated in the study. Parents who did not complete an assessment at day 30 for reasons other than a child’s death were excluded. Overall, the sample utilized in this study’s analyses consisted of 110 parents of 61 children with LLCs (Fig. 1).

Parent, family, and child characteristics are presented in Table 2. Among parents with foreign citizenship, 12 (48%) were German. The majority of parents not in employment at study entry were mothers (*n* = 13, 87%). Fifty-three families (87%) had supplementary insurance in addition to compulsory health insurance.

### Hospitalizations and LoS

**Fig. 2:**
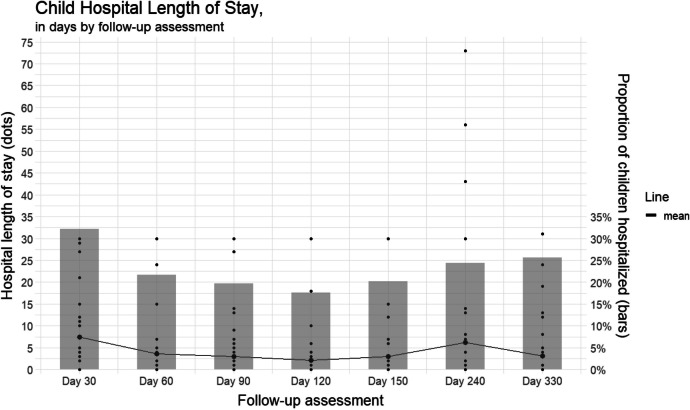
Child hospital LoS. Assessment periods were 30 days each for the first five assessments and 90 days each for the last two. The line gives the mean child hospital LoS. Each bar indicates the proportion of children hospitalized for at least 1 day for the corresponding assessment

Over the full study period, the median number of hospitalizations per child was 1 (interquartile range (IQR) 0–3, range (Rng.) 0–6). For the 30-day and 90-day assessment periods, the maximum number of hospitalizations per child was 2 and 3, respectively. Seventeen children (28%) had no hospitalization. The median total hospital LoS was 7 days (IQR 0–21, Rng. 0–227). Details on child hospital LoS are provided in Fig. 2.

### OOP non-medical expenses and financial support

**Fig. 3:**
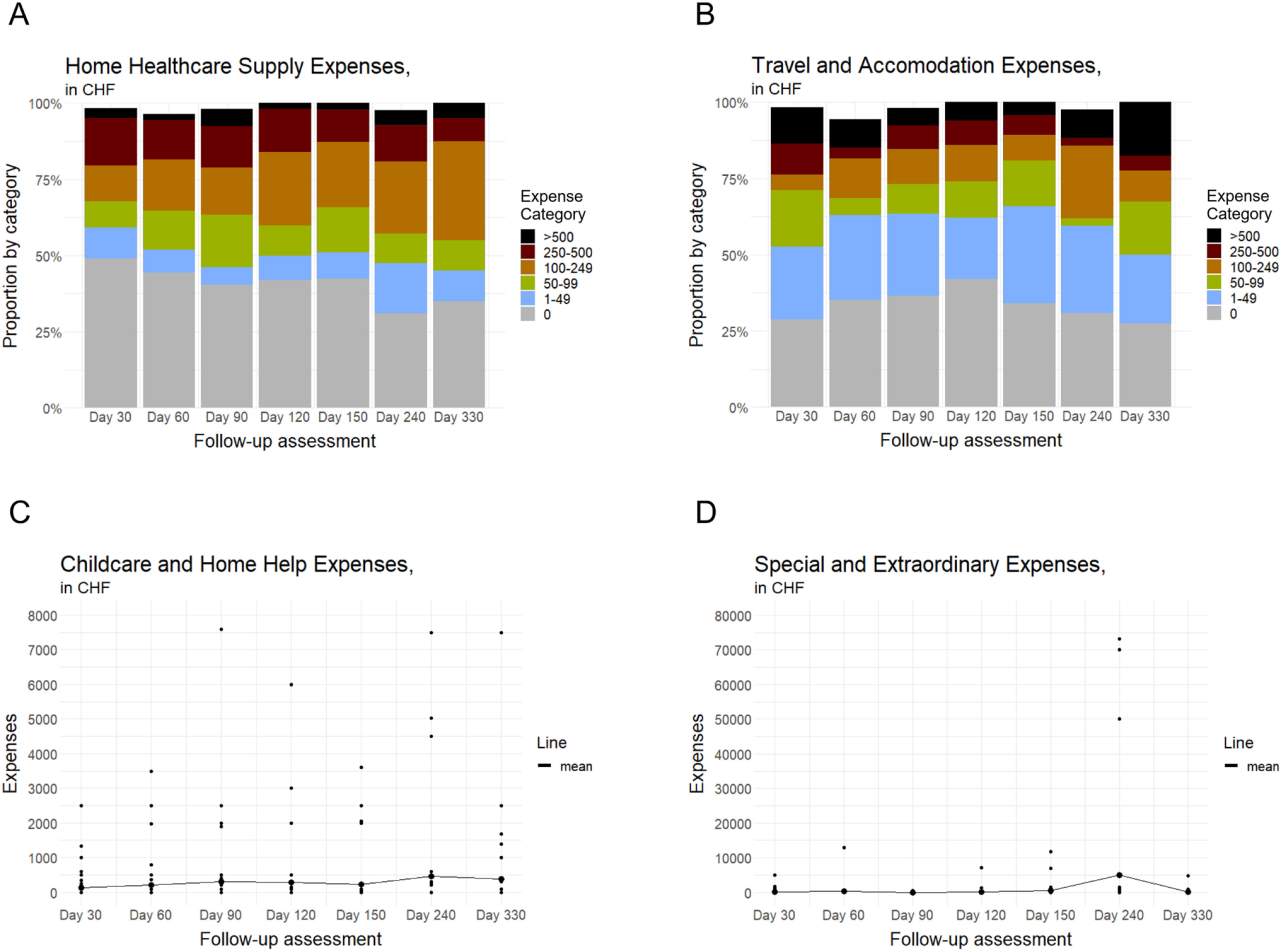
**A**–**D** Family OOP expenses. Assessment periods were 30 days each for the first five assessments and 90 days each for the last two. In subfigures **A** and **B**, the sum of the proportions is less than 100% due to missing data. CHF indicates Swiss francs

**Fig. 4:**
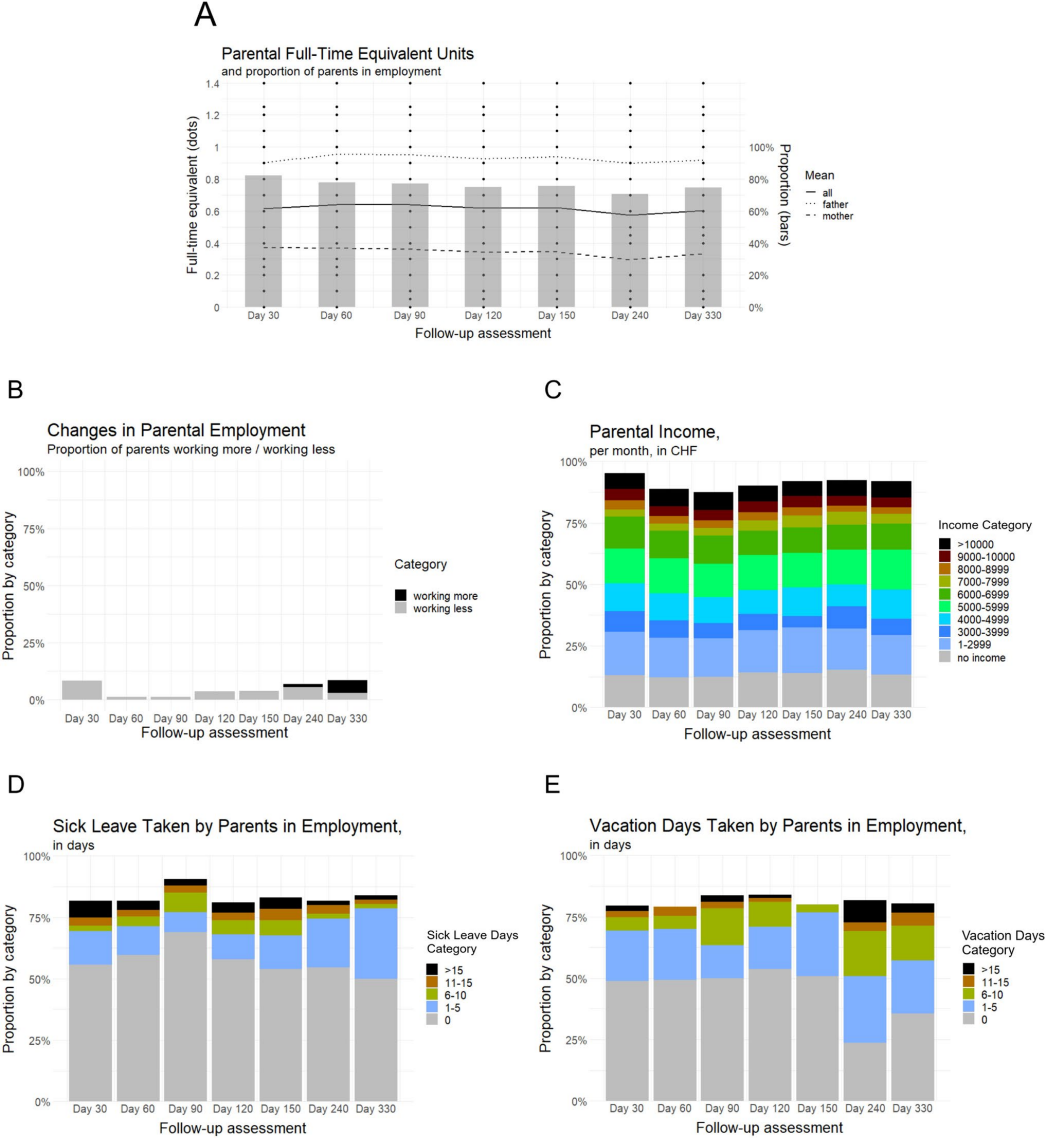
**A**–**E** Parental FTE, income, and work absences. Assessment periods were 30 days each for the first five assessments and 90 days each for the last two. The sum of the proportions is less than 100% due to missing data. Some parents reported > 1FTE, e.g., because of smaller side-line enterprises. CHF indicates Swiss francs

Figures 3A–D and 4A–E detail the outcomes of the 330-day follow-up. The proportion of families that did not incur any travel and accommodation expenses peaked at day 120 (Fig. 3B)—the same day the proportion of children hospitalized was lowest. Childcare and home help expenses averaged CHF480 per month for the 20 families (33%) that had such expenses (Fig. 3C). Throughout study participation, six families (10%) spent > CHF10,000 for special and extraordinary purchases (Fig. 3D). At each assessment, 50% or fewer families had financial support (Supplemental Table 5).

### Employment outcomes

The highest share of parents (*n* = 9, 8%) decreasing their individual FTE, primarily mothers (*n* = 7, 78%), was observed on day 30 (Fig. 4B). Throughout the 330-day follow-up, an average of 33% of mothers were not in employment, while for fathers, this applied to 5%.

### Missing data

The amount of missing data was highest for sick leave and vacation days (up to 21%) and lowest for OOP non-medical expenses (5% max.) (Supplemental Table 3). As for the latter, Little’s test showed that missing data were likely to be completely random (Supplemental Table 4).

### Bereavement outcomes

Sixteen children (26%) died during study participation. Deaths occurred at a median of 132 days (IQR 87–224, Rng. 6–252) after study entry. Locations of death were hospital (*n* = 8, 50%), home (*n* = 7, 44%), and other (*n* = 1, 6%). At the last follow-up assessment before death, five (19%) of the 26 participating parents of these 16 deceased children were not in employment (missing *n* = 3, 12%). Of the 23 parents (88%) who completed the bereavement assessment on day 120, five (22%) had increased their work commitments, while one (4%) reported a decrease. During the first 120 days of bereavement, four parents (17%) took > 30 days of sick leave, while two (9%) took > 30 vacation days. Parents increasing their work commitments during bereavement also reported an increase in income (Supplemental Table 6).

### Hospitalization-related implications

The seven care follow-up assessments provided 344 family and 633 parent observations for use in the regression analyses. The adjusted fixed-effects analyses showed a positive association of child hospital LoS with travel and accommodation expenses (coefficient 4.18, 95% confidence interval 2.20–6.16). For every 1-day increase in hospital LoS, travel and accommodation expenses increased by 6.2% compared to the reference group. The median hospital LoS was 7 days (IQR 0–21, Rng. 0–227). With a mean difference in travel and accommodation expenses of CHF4.18, families spent an additional CHF29 for every week of their child’s hospitalization. No other associations were found between child hospital LoS and our other outcomes (Table 3).

**Table 3:** Crude and adjusted two-way linear fixed-effects models of the effect of child hospital LoS on family/parent economic outcomes

Outcome	Crude models^a^				Adjusted models^b^					
	Observations^e^	Coef.	[95% CI]	*p*	Observations^e^	Coef.	[95% CI]	*p*	Mean reference group^f^	Relative effect^g^
Out-of-pocket expenses										
Home health-care supplies^c^	339	− 1.33	[− 3.37 to 0.70]	0.20	329	− 1.29	[− 3.33 to 0.74]	0.21	181.50	− 0.7%
Travel and accommodation^c^	338	4.46	[2.52 to 6.41]	< 0.001	328	4.18	[2.20 to 6.16]	< 0.001	67.76	6.2%
Childcare and home help^c^	339	− 3.40	[− 15.52 to 8.72]	0.58	329	− 3.92	[− 12.77 to 4.93]	0.39	542.33	− 0.7%
Special and extraordinary^c^	338	− 46.01	[− 156.71 to 64.69]	0.42	328	− 52.38	[− 165.67 to 60.91]	0.37	1843.99	− 2.8%
Employment and income										
Full-time equivalent unit	600	− 0.00	[− 0.00 to 0.00]	0.99	585	0.00	[− 0.00 to 0.00]	0.92	0.60	0.0%
Income^c^	577	1.69	[− 7.73 to 11.11]	0.73	564	2.90	[− 6.74 to 12.54]	0.56	4822.83	0.1%
Work absenteeism										
Sick leave^d^	404	0.03	[− 0.02 to 0.08]	0.30	401	0.03	[− 0.02 to 0.08]	0.27	2.02	1.5%
Vacation^d^	393	− 0.06	[− 0.13 to 0.01]	0.10	388	− 0.06	[− 0.14 to 0.01]	0.08	2.23	− 2.7%

*Coef.* coefficient, *CI* confidence interval

Estimated coefficients represent mean differences in outcomes per day of hospitalization

^a^The crude models are linear fixed-effects models with time- and subject-fixed effects

^b^The adjusted models are linear fixed-effects models with time- and subject-fixed-effects adjusted for financial support and follow-up assessment period length

^c^In Swiss francs

^d^In days

^e^Number of observations varies because of variation in missing outcome and financial support data (see Supplemental Tables for information on missing data)

^f^The reference group used as a benchmark included all children who did not have any hospital stay during study participation

^g^Relative effect = (estimated coefficient of the adjusted model/mean expenses reference group) × 100

### Robustness check

The complete-case and subgroup analyses support the findings of our fixed-effects analyses, with two exceptions: Hospital LoS was no longer associated with travel and accommodation expenses for families with a household income of ≥ CHF100,000 and those with a home-to-hospital travel distance of ≤ 20 km (Supplemental Tables 7–12).

## Discussion

In a sample of parents of children with a LLC, we investigated the financial and employment implications of two events: child hospitalization and death. Child hospitalization, i.e., hospital LoS, was positively associated with families’ travel and accommodation expenses. During the first 120 days of bereavement, more than one-fifth of grieving parents increased their work commitments.

The additional travel and accommodation expenses endured by families are likely explained by parents maintaining a bedside presence during their child’s hospitalization, e.g., for transportation, parking, and board and lodging [22–24, 26]. In our sample, the extra travel and accommodation expenses incurred by parents per week of hospitalization were rather low. For some parents, setting-specific factors such as parking and food vouchers or free-of-charge hospital accommodation for parents may have limited these expenses. However, in settings with less support, these expenses could be much higher. Moreover, with repeated and lengthy hospitalizations, travel and accommodation expenses are likely to be greater.

Consistent with previous research, our analyses indicate that families residing farther away from the hospital experience increased travel and accommodation expenses [22, 24]. Families with lower income may have reported their spending more accurately, as they were required to provide detailed records to financial assistance programs. This may explain the association of hospital LoS with travel and accommodation expenses for these families. Another explanation could be that, due to affordable housing options, families with lower income live farther away from hospitals.

There are several possible explanations for the lack of association between hospital LoS and other OOP non-medical expenses. First, during their child’s hospitalization, a family may purchase home healthcare supplies in preparation for their child’s hospital-to-home transition. Being prepared in terms of medication, equipment, and supplies has been shown to be a priority for families [42]. Second, informal care provided by relatives and friends may explain both the low number of families that incurred formal childcare and home help expenses and the lack of association between such expenses and hospital LoS. Here, our findings contrast with previous research, which suggested that childcare expenses, e.g., for siblings, would normally increase during hospitalizations [22, 23]. Cultural differences may have an influence on families’ informal caregiving arrangements [43]. Third, a child’s functional limitations, namely, their dependency on medical technology and equipment, may be a stronger explanatory factor for increased special and extraordinary expenses than hospitalization [12]. Although few families in our study incurred such expenses, for those who did, they were exceedingly high.

Contrary to our expectations, we did not find any significant hospitalization-related employment or income implications. Income replacement mechanisms, e.g., paid sick leave, and flexible employment arrangements, e.g., working from home, may have protected families from adverse effects. Previous research suggests that income loss is less severe in parents with flexible work arrangements [23]. As a contextual matter, it is worth noting that data collection occurred during the coronavirus disease 2019 pandemic, which brought a substantial increase in flexible work practices [44].

In addition, families were recruited a median of 1 year after their child’s diagnosis, when certain employment adaptations may already have taken place. For instance, mothers of children diagnosed during maternity leave may have extended that leave. A previous study of employment implications in families of children with special healthcare needs found that forgone employment was disproportionately high among mothers of young children (0–5 years) [45]. Compared to mothers in the general population, those in our study were less likely to be employed. Across the care follow-up assessments, an average of 33% of participating mothers did not engage in employment, compared with 17% in the general population [46].

Regarding the bereavement follow-up, parents’ increasing work commitments may have been motived by both economic considerations and a desire to resume work, e.g., as a means of distraction [33, 47]. That is, the observed increases in work commitment do not necessarily indicate full readiness or restored functionality [33]. In general pediatrics, parental grief-related costs associated with work-presenteeism outweighed those associated with work-absenteeism [33]. The same principle may also explain a number of our bereavement follow-up observations of parental income, sick leave, and vacation days.

One of this study’s major strengths is its longitudinal design: multiple assessments provided us with a realistic representation of how LLC-related events—and costs—develop over time. Another is its fixed-effects approach, which controls for time-invariant heterogeneity and confounders. Nevertheless, it also has notable limitations, including the relatively small sample size and limited number of hospitalizations, which limit statistical power and generalizability. In addition, sample bias cannot be ruled out, as families experiencing high psychological, emotional, social, or financial burdens may have been more likely to decline study participation. This effect might have been accentuated by additional attrition bias during the study. Moreover, outcome data were self-reported, leaving them vulnerable to response and recall bias. Some parents may have been reluctant to disclose their income. However, our instruments’ discrete response categories and primarily monthly assessments would have minimized this type of bias. Finally, hospitalization rates may have been influenced by the coronavirus disease 2019 pandemic.

## Conclusions

Hospitalization-related travel and accommodation expenses exacerbate the often substantial financial burden experienced by families of children with LLCs. To protect these families’ financial well-being, adequate financial support should be made available to them. Where such financial support currently exists, additional efforts should be made both to enhance affected parents’ awareness and assist them with the application processes. Future research is also needed to further explore and explain the effect of LLCs in children on their parents’ employment, e.g., by comparing families of children with a LLC to those of children without a LLC. To date, little is known of the factors behind these parents’ decisions to reduce or increase their work commitments through their children’s illness and their own bereavement.

## Supplementary Information

Below is the link to the electronic supplementary material.

Supplementary file1 (PDF 592 KB)

## Data Availability

Deidentified individual participant data (including data dictionaries) will be made available, in addition to study protocols, the statistical analysis plan, and the informed consent form. The data will be made available upon publication to researchers who provide a methodologically sound proposal for use in achieving the goals of the approved proposal. Proposals should be submitted to Karin Zimmermann (karin.zimmermann@unibas.ch).

## Abbreviations

CHF: Swiss francs
CI: Confidence interval
Coef.: Coefficient
FTE: Full-time equivalent
IQR: Interquartile range
LLC: Life-limiting condition
LoS: Length of stay
OOP: Out-of-pocket
Rng.: Range
SD: Standard deviation
SPPC: Specialized pediatric palliative care

## References

[1] Together for Short Lives (2018) A guide to children’s palliative care: supporting babies, children and young people with life-limiting and life-threatening conditions and their families. https://www.togetherforshortlives.org.uk/app/uploads/2018/03/TfSL-A-Guide-to-Children%E2%80%99s-Palliative-Care-Fourth-Edition-5.pdf. Accessed 09 March 2023

[2] Fraser LK, Murtagh FE, Aldridge J, Sheldon T, Gilbody S, Hewitt C (2021) Health of mothers of children with a life-limiting condition: a comparative cohort study. Arch Dis Child. 106(10):987–993. 10.1136/archdischild-2020-320655 33653713 10.1136/archdischild-2020-320655 PMC8461446

[3] Fisher V, Atkin K, Fraser LK (2022) The health of mothers of children with a life-limiting condition: a qualitative interview study. Palliat Med. 36(9):1418–1425. 10.1177/02692163221122325 36113084 10.1177/02692163221122325 PMC9597138

[4] Fisher V, Fraser L, Taylor J (2021) Experiences of fathers of children with a life-limiting condition: a systematic review and qualitative synthesis. BMJ Support Palliat Care. 13(1):15–26. 10.1136/bmjspcare-2021-003019 34140322 10.1136/bmjspcare-2021-003019 PMC9985706

[5] Courtney E, Kiernan G, Guerin S, Ryan K, McQuillan R (2018) Mothers’ perspectives of the experience and impact of caring for their child with a life-limiting neurodevelopmental disability. Child Care Health Dev. 44(5):704–710. 10.1111/cch.12580 29938823 10.1111/cch.12580

[6] Nicholas DB, Beaune L, Barrera M, Blumberg J, Belletrutti M (2016) Examining the experiences of fathers of children with a life-limiting illness. J Soc Work End Life Palliat Care. 12(1–2):126–144. 10.1080/15524256.2016.1156601 27143577 10.1080/15524256.2016.1156601

[7] Randall DC (2017) Two futures: financial and practical realities for parents of living with a life limited child. Compr Child Adolesc Nurs. 40(4):257–267. 10.1080/24694193.2017.1376360 29125321 10.1080/24694193.2017.1376360

[8] Carlton EF, Becker NV, Moniz MH, Scott JW, Prescott HC, Chua KP (2023) Out-of-pocket spending for non-birth-related hospitalizations of privately insured US children, 2017 to 2019. JAMA Pediatr. 177(5):516–525. 10.1001/jamapediatrics.2023.0130 36972040 10.1001/jamapediatrics.2023.0130 PMC10043803

[9] Bassett HK, Coller RJ, Beck J, Hummel K, Tiedt KA, Flaherty B, et al (2020) Financial difficulties in families of hospitalized children. J Hosp Med. 15(11): 652-658. 10.12788/jhm.3500 10.12788/jhm.3500 33147127

[10] Elhoff JJ, McHugh KE, Buckley JR, Morris SA, Simpson KN, Scheurer MA (2018) Out-of-pocket medical expenses in severe CHD. Cardiol Young. 28(8):1014–1018. 10.1017/s1047951118000768 29923476 10.1017/S1047951118000768

[11] Tsimicalis A, Stevens B, Ungar WJ, McKeever P, Greenberg M (2011) The cost of childhood cancer from the family’s perspective: a critical review. Pediatr Blood Cancer. 56(5):707–717. 10.1002/pbc.22685 21370401 10.1002/pbc.22685

[12] Bourke-Taylor H, Cotter C, Stephan R (2014) Young children with cerebral palsy: families self-reported equipment needs and out-of-pocket expenditure. Child Care Health Dev. 40(5):654–662. 10.1111/cch.12098 23952344 10.1111/cch.12098

[13] McClung N, Glidewell J, Farr SL (2018) Financial burdens and mental health needs in families of children with congenital heart disease. Congenit Heart Dis. 13(4):554–562. 10.1111/chd.12605 29624879 10.1111/chd.12605 PMC6105538

[14] Bona K, London WB, Guo D, Frank DA, Wolfe J (2016) Trajectory of material hardship and income poverty in families of children undergoing chemotherapy: a prospective cohort study. Pediatr Blood Cancer. 63(1):105–111. 10.1002/pbc.25762 26398865 10.1002/pbc.25762

[15] Tsimicalis A, Stevens B, Ungar WJ, McKeever P, Greenberg M, Agha M et al (2013) A mixed method approach to describe the out-of-pocket expenses incurred by families of children with cancer. Pediatr Blood Cancer 60(3):438–445. 10.1002/pbc.24324 23015413 10.1002/pbc.24324

[16] Bally JMG, Smith NR, Holtslander L, Duncan V, Hodgson-Viden H, Mpofu C et al (2018) A metasynthesis: uncovering what is known about the experiences of families with children who have life-limiting and life-threatening illnesses. J Pediatr Nurs 38:88–98. 10.1016/j.pedn.2017.11.004 29357986 10.1016/j.pedn.2017.11.004

[17] Jaaniste T, Cuganesan A, Chin WLA, Tan SC, Coombs S, Heaton M et al (2022) Living with a child who has a life-limiting condition: the functioning of well-siblings and parents. Child Care Health Dev. 48(2):269–276. 10.1111/cch.12927 34766366 10.1111/cch.12927

[18] Boyden JY, Hill DL, Nye RT, Bona K, Johnston EE, Hinds P et al (2022) Pediatric palliative care parents’ distress, financial difficulty, and child symptoms. J Pain Symptom Manage. 63(2):271–282. 10.1016/j.jpainsymman.2021.08.004 34425212 10.1016/j.jpainsymman.2021.08.004 PMC8816828

[19] Smith S, Tallon M, Clark C, Jones L, Mörelius E (2022) “You never exhale fully because you’re not sure what’s next”: parents’ experiences of stress caring for children with chronic conditions. Front Pediatr. 10:902655. 10.3389/fped.2022.902655 35832577 10.3389/fped.2022.902655 PMC9271768

[20] Kim CH, Song IG, Kim MS, Lee JY, Lim NG, Shin HY (2020) Healthcare utilization among children and young people with life-limiting conditions: exploring palliative care needs using National Health Insurance claims data. Sci Rep. 10(1):2692. 10.1038/s41598-020-59499-x 32060337 10.1038/s41598-020-59499-x PMC7021730

[21] Fraser LK, Parslow R (2018) Children with life-limiting conditions in paediatric intensive care units: a national cohort, data linkage study. Arch Dis Child. 103(6):540–547. 10.1136/archdischild-2017-312638 28705790 10.1136/archdischild-2017-312638 PMC5965357

[22] DiFazio RL, Vessey JA (2013) Non-medical out-of-pocket expenses incurred by families during their child’s hospitalization. J Child Health Care. 17(3):230–241. 10.1177/1367493512461459 23711489 10.1177/1367493512461459

[23] Chang LV, Shah AN, Hoefgen ER, Auger KA, Weng H, Simmons JM et al (2018) Lost earnings and nonmedical expenses of pediatric hospitalizations. Pediatrics. 142(3):e20180195. 10.1542/peds.2018-0195 30104421 10.1542/peds.2018-0195 PMC12747580

[24] Mumford V, Baysari MT, Kalinin D, Raban MZ, McCullagh C, Karnon J et al (2018) Measuring the financial and productivity burden of paediatric hospitalisation on the wider family network. J Paediatr Child Health. 54(9):987–996. 10.1111/jpc.13923 29671913 10.1111/jpc.13923 PMC6635734

[25] Clark ME, Cummings BM, Kuhlthau K, Frassica N, Noviski N (2019) Impact of pediatric intensive care unit admission on family financial status and productivity: a pilot study. J Intensive Care Med. 34(11–12):973–977. 10.1177/0885066617723278 28797189 10.1177/0885066617723278

[26] Wasserfallen JB, Bossuat C, Perrin E, Cotting J (2006) Costs borne by families of children hospitalized in a pediatric intensive care unit: a pilot study. Swiss Med Wkly. 136(49–50):800–804. 10.4414/smw.2006.11585 17299658 10.4414/smw.2006.11585

[27] Lykke C, Ekholm O, Schmiegelow K, Olsen M, Sjøgren P (2019) Anxiety and depression in bereaved parents after losing a child due to life-limiting diagnoses: a Danish nationwide questionnaire survey. J Pain Symptom Manage. 58(4):596–604. 10.1016/j.jpainsymman.2019.06.025 31276811 10.1016/j.jpainsymman.2019.06.025

[28] Vig PS, Lim JY, Lee RWL, Huang H, Tan XH, Lim WQ et al (2021) Parental bereavement - impact of death of neonates and children under 12 years on personhood of parents: a systematic scoping review. BMC Palliat Care. 20(1):136. 10.1186/s12904-021-00831-1 34481491 10.1186/s12904-021-00831-1 PMC8418708

[29] Dias N, Brandon D, Haase JE, Tanabe P (2018) Bereaved parents’ health status during the first 6 months after their child’s death. Am J Hosp Palliat Care. 35(6):829–839. 10.1177/1049909117744188 29202599 10.1177/1049909117744188

[30] Youngblut JM, Brooten D, Cantwell GP, del Moral T, Totapally B (2013) Parent health and functioning 13 months after infant or child NICU/PICU death. Pediatrics. 132(5):e1295-1301. 10.1542/peds.2013-1194 24101760 10.1542/peds.2013-1194 PMC3813397

[31] Hawthorne DM, Youngblut JM, Brooten D (2016) Parent spirituality, grief, and mental health at 1 and 3 months after their infant’s/child’s death in an intensive care unit. J Pediatr Nurs. 31(1):73–80. 10.1016/j.pedn.2015.07.008 26320884 10.1016/j.pedn.2015.07.008 PMC4975148

[32] Butler AE, Hall H, Copnell B (2018) Bereaved parents’ experiences of research participation. BMC Palliat Care. 17(1):122. 10.1186/s12904-018-0375-4 30404631 10.1186/s12904-018-0375-4 PMC6223065

[33] Fox M, Cacciatore J, Lacasse JR (2014) Child death in the United States: productivity and the economic burden of parental grief. Death Stud. 38(6–10):597–602. 10.1080/07481187.2013.820230 24588841 10.1080/07481187.2013.820230

[34] Zimmermann K, Simon M, Scheinemann K, Tinner Oehler EM, Widler M et al (2022) Specialised Paediatric PAlliativE CaRe: assessing family, healthcare professionals and health system outcomes in a multi-site context of various care settings: SPhAERA study protocol. BMC Palliat Care 21(1):188. 10.1186/s12904-022-01089-x 36324132 10.1186/s12904-022-01089-x PMC9628037

[35] McDonald P, Bradley L, Brown K (2008) Visibility in the workplace: still an essential ingredient for career success? Int. J. Hum. Resour. Manag. 19(12):2198–2215. 10.1080/09585190802479447

[36] Roderick JAL (1988) A test of missing completely at random for multivariate data with missing values. J AM Stat Assoc. 83(404): p. 1198-1202. http://www.jstor.org/stable/2290157. Accessed 29 June 2023

[37] Stock JH and Watson MW (2012) Introduction to econometrics. International edition ed. Vol. 3rd Edition., United Kingdom: Peason Education

[38] Gunasekara FI, Richardson K, Carter K, Blakely T (2014) Fixed effects analysis of repeated measures data. Int J Epidemiol 43(1):264–269. 10.1093/ije/dyt221 24366487 10.1093/ije/dyt221

[39] R Core Team (2018) R: a language and environment for statistical computing, R.F.f.S. Computing, Editor., R Foundation for Statistical Computing: Vienna, Austria. https://www.R-project.org

[40] Croissant Y and Millo G (2008) Panel Data Econometrics in R: The plm Package. Journal of Statistical Software. 27(2): p. 1-43. https://www.jstatsoft.org/index.php/jss/article/view/v027i02. Accessed 12 October 2023

[41] Hausman JA (1978) Specification tests in econometrics. Econometrica. 46(6): p 1251-1271. http://www.jstor.org/stable/1913827. Accessed 28 June 2023

[42] Leyenaar JK, O’Brien ER, Leslie LK, Lindenauer PK, Mangione-Smith RM (2017) Families’ priorities regarding hospital-to-home transitions for children with medical complexity. Pediatrics. 139(1):e20161581. 10.1542/peds.2016-1581 27940509 10.1542/peds.2016-1581 PMC5192089

[43] Bauer J, Sousa-Poza A (2015) Impacts of informal caregiving on caregiver employment, health, and family. Population Ageing. 8:113–145. 10.1007/s12062-015-9116-0

[44] Brynjolfsson E, Horton JJ, Ozimek A, Rock D, Sharma G, and TuYe H-Y (2020) COVID-19 and remote work: an early look at US data. No. 27344: p 3-4. https://www.nber.org/system/files/working_papers/w27344/w27344.pdf. Accessed 20 August 20 2023

[45] Foster CC, Chorniy A, Kwon S, Kan K, Heard-Garris N, Davis MM (2021) Children with special health care needs and forgone family employment. Pediatrics. 148(3):e2020035378. 10.1542/peds.2020-035378 34433691 10.1542/peds.2020-035378 PMC9219960

[46] Bundesamt für Statistik (2023) Erwerbsquoten nach Geschlecht und Familiensituation 2010-2022: Tabelle. Available from: https://www.bfs.admin.ch/bfs/de/home/statistiken/wirtschaftliche-soziale-situation-bevoelkerung/gleichstellung-frau-mann/erwerbstaetigkeit/erwerbsbeteiligung.assetdetail.25605564.html. Accessed 13 August 2023

[47] Pedraza EC, Michel G, Altherr A, Hendriks MJ, De Clercq E (2023) Coping strategies in families who lost a child to cancer: a scoping review. EJC Paediatric Oncology. 1:100011. 10.1016/j.ejcped.2023.100011

[48] Bundesamt für Statistik. Haushaltseinkommen und- ausgaben nach Haushaltstyp: Tabelle. Available from: https://www.bfs.admin.ch/bfs/de/home/statistiken/wirtschaftliche-soziale-situation-bevoelkerung/einkommen-verbrauch-vermoegen/haushaltsbudget.assetdetail.20024372.html. Accessed 15 December 2023

