# Supplementary material for: Hospitalization- and death-related financial and employment effects in parents of children with life-limiting conditions: a fixed-effects analysis
Source: Eur J Pediatr. 2024 Jul 10;183(10):4215–27. doi: 10.1007/s00431-024-05680-7 (PMC11413104; doi:10.1007/s00431-024-05680-7)
Supplement: Supplementary file 1 — Supplementary file1 (PDF 592 KB) [file 431_2024_5680_MOESM1_ESM.pdf]

Supplemental materials

Journal: European Journal of Pediatrics

Article Title: Hospitalization- and death-related financial and employment effects in parents of children with life-limiting conditions: a fixed-effects analysis

Authors: Stefan Mitterer[1], MPhil HE, Karin Zimmermann[1,2], PhD, RN, Günther Fink3, PhD, Michael Simon[1], PhD, RN, Anne-Kathrin Gerber[1], MA HS, RN, Eva Bergsträsser[2], MD, MSc Palliative Medicine

Affiliations: [1]Institute of Nursing Science, Department of Public Health, University of Basel, Basel, Switzerland; [2]Paediatric Palliative Care and Children's Research Center, University Children's Hospital Zurich, Zurich, Switzerland; and [3]Swiss Tropical and Public Health Institute, University of Basel, Basel, Switzerland

Corresponding Author: Karin Zimmermann, Institute of Nursing Science, Department of Public Health, University of Basel, Bernoullistrasse. 28, CH-4065 Basel [karin.zimmermann@unibas.ch], +41 (0)61 207 61 05.

Supplemental Material 1: Empirical model, statistical analysis and robustness check

Empirical model:

$$\boldsymbol{Y_{it} = \alpha_i + \beta_1 LoS_{it} + \ldots + \beta_k X_{k,it} + \lambda_t + \varepsilon_{it}}$$

Where **Y** represents the economic outcome of interest in a subject (i.e., family/parent) **i** at time **t**. The coefficient **β** can be interpreted as the within-subject effect on outcome Y of having been exposed to child hospital LoS ($\mathbf{LoS_{it}}$). The subject fixed-effects are given by $\boldsymbol{\alpha_i}$, representing the subject-specific intercepts (α_1, …, α_n). The time fixed-effects are given by $\boldsymbol{\lambda_t}$, representing the intercept of each time period. $\mathbf{X_{2,it}}$, …, $\mathbf{X_{k,it}}$ are other observed determinants of Y that are correlated with LoS_{it} and that vary across subjects and over time, e.g., financial support. $\boldsymbol{\varepsilon_{it}}$ is the unobserved error term.

Statistical analysis:

By including subject- and time-fixed effects, fixed-effects models implicitly control for variables that are constant over time but differ across subjects, as well as for those variables that are constant across subjects but change over time [37, 38]. This controls for any time-invariant factors that may have confounding effects or increase the data's heterogeneity [37, 38]. As financial support varies with time and between subjects, we included this variable in our adjusted models. In addition, a dichotomous indicator for period length, i.e., short (30 days) or long (90 days), was added to account for the differences in observation length. In the parent-level analyses, a child-fixed effect was included to account for within-family correlations. For all analyses, cluster-robust standard errors were used. For the regression analyses, categorical outcome variables were converted to continuous variables by assigning the midpoint of each categorical range as the representative value and using a conservative estimate of the categories' lower limit +1 for open-ended categories. In presenting the regression outputs, our reference group—consisting of all children who did not have any hospital stay during study participation—functioned as a benchmark.

Robustness check:

Although both the fixed-effects and the random-effects models were consistent, we chose to report the former because of their ability to control for time-invariant unobserved heterogeneity and omitted variables. The results of the random-effects models are presented in Supplemental Table 2.

To check whether our results are robust regarding attrition and death, a binary variable indicating full 330-day study participation was created. To test for the possibility that limited exposure and high income could mitigate potential effects, we specified subgroups based respectively on total hospital LoS and household income. Also, because the association of hospital LoS with both home healthcare supply expenses and special and extraordinary expenses could vary by diagnosis, we created dichotomous diagnosis-specific subgroups, i.e., neurological vs. non-neurological. The association between hospital LoS and travel and accommodation expenses was tested based on home-to-hospital travel distance.

Supplemental Table 1: Results of the Hausman test.

Outcome variable	Adjusted fixed-effects model vs. adjusted random-effects model
	p
**Out-of-pocket expenses**	
Home healthcare supplies	*0.30*[a]
Travel and accommodation	*0.94*[a]
Childcare and home help	*0.86*[a]
Special and extraordinary	*0.28*[a]
**Employment and income**	
Full-time equivalent unit	*0.09*[a]
Income	*0.32*[a]
**Work absenteeism**	
Sick leave days	*0.47*[a]
Vacation days	*0.06*[a]

[a] A p-value of >0.05 suggests that there is no significant evidence to reject the null hypothesis that the fixed-effects model is preferred, indicating that both models are consistent.

Supplemental Table 2: Crude and adjusted random-effects models of the effect of a child's hospital LoS on family/parent economic outcomes.

Outcome	Crude models[a]				Adjusted models[b]					
	Observations[e]	Coef.	[95% CI]	*p*	Observations[e]	Coef.	[95% CI]	*p*	Mean reference group[f]	Relative effect[g]
**Out-of-pocket expenses**										
Home healthcare supplies[c]	339	-2.07	[-3.73 to -0.42]	*0.01*	329	-2.10	[-3.73 to -0.47]	*0.01*	181.50	-1.2%
Travel and accommodation[c]	338	4.44	[2.75 to 6.13]	*<0.001*	328	4.21	[2.51 to 5.91]	*<0.001*	67.76	6.2%
Childcare and home help[c]	339	-4.55	[-14.93 to 5.84]	*0.39*	329	-4.92	[-12.51 to 2.68]	*0.20*	542.33	-0.9%
Special and extraordinary[c]	338	-25.02	[-93.24 to 43.20]	*0.47*	328	-26.28	[-94.62 to 42.06]	*0.45*	1843.99	-1.4%
**Employment and income**										
Full-time equivalent unit	600	-0.00	[-0.00 to 0.00]	*0.67*	585	0.00	[-0.00 to 0.00]	*0.93*	0.60	0.0%
Income[c]	577	1.39	[-7.67 to 10.45]	*0.76*	564	3.18	[-6.10 to 12.46]	*0.50*	4822.83	0.1%
**Work absenteeism**										
Sick leave[d]	404	0.03	[-0.01 to 0.08]	*0.16*	401	0.04	[-0.01 to 0.08]	*0.14*	2.02	2.0%
Vacation[d]	393	-0.00	[-0.06 to 0.05]	*0.95*	388	-0.01	[-0.06 to 0.04]	*0.76*	2.23	-0.4%

Coef. indicates coefficient; CI, confidence interval.

Note: Estimated coefficients represent mean differences in outcomes per day of hospitalization.

[a] The crude models are random-effects models with time- and subject-fixed effects.

[b] The adjusted models are random-effects models with time- and subject-fixed effects adjusted for financial support and follow-up assessment period length.

[c] in Swiss francs.

[d] in days.

[e] Number of observations vary because of variation in missing outcome and financial support data (see Supplemental Tables 3 and 5 for information on missing data).

[f] The reference group used as a benchmark included all children who did not have any hospital stay during study participation.

[g] Relative effect = (estimated coefficient of the adjusted model / mean expenses reference group) x 100.

Supplemental Table 3: Outcome data of the 330-day care follow-up assessment.

Outcome	Day 30	Day 60	Day 90	Day 120	Day 150	Day 240	Day 330
**Out-of-pocket expenses, in CHF**	**Families n=59**	**Families n=54**	**Families n=52**	**Families n=50**	**Families n=47**	**Families n=42**	**Families n=40**
Home healthcare supplies, n (%)							
0	29 (49%)	24 (44%)	21 (40%)	21 (42%)	20 (43%)	13 (31%)	14 (35%)
1–49	6 (10%)	4 (7%)	3 (6%)	4 (8%)	4 (8%)	7 (17%)	4 (10%)
50–99	5 (9%)	7 (13%)	9 (17%)	5 (10%)	7 (15%)	4 (9%)	4 (10%)
100–249	7 (12%)	9 (17%)	8 (15%)	12 (24%)	10 (21%)	10 (24%)	13 (33%)
250–500	9 (15%)	7 (13%)	7 (14%)	7 (14%)	5 (11%)	5 (12%)	3 (7%)
>500	2 (3%)	1 (2%)	3 (6%)	1 (2%)	1 (2%)	2 (5%)	2 (5%)
missing	*1 (2%)*	*2 (4%)*	*1 (2%)*	-	-	*1 (2%)*	-
Travel and accommodation, n (%)							
0	17 (29%)	19 (35%)	19 (37%)	21 (42%)	16 (34%)	13 (31%)	11 (27%)
1–49	14 (24%)	15 (28%)	14 (27%)	10 (20%)	15 (32%)	12 (29%)	9 (23%)
50–99	11 (18%)	3 (6%)	5 (10%)	6 (12%)	7 (15%)	1 (2%)	7 (18%)
100–249	3 (5%)	7 (13%)	6 (12%)	6 (12%)	4 (9%)	10 (24%)	4 (10%)
250–500	6 (10%)	2 (4%)	4 (8%)	4 (8%)	3 (6%)	1 (2%)	2 (5%)
>500	7 (12%)	5 (9%)	3 (6%)	3 (6%)	2 (4%)	4 (10%)	7 (18%)
missing	*1 (2%)*	*3 (6%)*	*1 (2%)*	-	-	*1 (2%)*	-
Childcare and home help							
Mean (SD)	137.1 (414.9)	199.8 (649.4)	307.8 (1'161.9)	289.0 (1'002.4)	223.2 (739.0)	459.5 (1'531.4)	387.3 (1'268.2)
Median (IQR)	0 (0–0)	0 (0–0)	0 (0–0)	0 (0–0)	0 (0–0)	0 (0–0)	0 (0–25)
Range	0–2'500	0–3'500	0–7'600	0–6'000	0–3'600	0–7'500	0–7'500
missing, no (%)	*1 (2%)*	*2 (4%)*	*1 (2%)*	-	-	*1 (2%)*	-
Special and extraordinary							
Mean (SD)	211.5 (725.7)	297.1 (1801.0)	23.9 (93.3)	178.8 (1'017.6)	464.7 (1'976.2)	4'903.9 (17'398.3)	197.5 (774.8)
Median (IQR)	0 (0–0)	0 (0–0)	0 (0–0)	0 (0–0)	0 (0–0)	0 (0–0)	0 (0–0)
Range	0–5'000	0–13'000	0–500	0–7'090	0–11'800	0–73'240	0–4'800
missing, no (%)	*1 (2%)*	*2 (4%)*	*1 (2%)*	-	-	*2 (5%)*	-
**Employment and income**	**Parents n=107**	**Parents n=99**	**Parents n=96**	**Parents n=92**	**Parents n=86**	**Parents n=78**	**Parents n=75**

Outcome	Day 30	Day 60	Day 90	Day 120	Day 150	Day 240	Day 330
Full-time equivalent unit							
Mean (SD)	0.6 (0.4)	0.6 (0.4)	0.6 (0.4)	0.6 (0.4)	0.6 (0.4)	0.6 (0.4)	0.6 (0.4)
Median (IQR)	0.6 (0.2–1.0)	0.8 (0.3–1.0)	0.8 (0.3–1.0)	0.7 (0.2–1.0)	0.8 (0.2–1.0)	0.6 (0.1–1.0)	0.7 (0.2–1.0)
Range	0–1.4	0–1.4	0–1.4	0–1.4	0–1.4	0–1.4	0–1.4
missing, n (%)	*1 (1%)*	*7 (7%)*	*7 (7%)*	*5 (5%)*	*4 (5%)*	*5 (6%)*	*4 (5%)*
Income in CHF, n (%)							
No income	14 (13%)	12 (12%)	12 (13%)	13 (14%)	12 (14%)	12 (15%)	10 (13%)
1–2’999	19 (18%)	16 (16%)	15 (16%)	16 (17%)	16 (19%)	13 (17%)	12 (16%)
3’000–3’999	9 (8%)	7 (7%)	6 (6%)	6 (7%)	4 (5%)	7 (9%)	5 (7%)
4’000–4’999	12 (11%)	11 (11%)	10 (10%)	9 (10%)	10 (12%)	7 (9%)	9 (12%)
5’000–5’999	15 (14%)	14 (14%)	13 (14%)	13 (14%)	12 (14%)	11 (14%)	12 (16%)
6’000–6’999	14 (13%)	11 (11%)	11 (11%)	9 (10%)	9 (10%)	8 (10%)	8 (11%)
7’000–7’999	3 (3%)	3 (3%)	3 (3%)	4 (4%)	4 (5%)	4 (5%)	3 (4%)
8’000–8’999	4 (4%)	3 (3%)	3 (3%)	3 (3%)	3 (3%)	2 (3%)	2 (3%)
9’000–10’000	5 (5%)	4 (4%)	4 (4%)	4 (4%)	4 (5%)	3 (4%)	3 (4%)
>10’000	7 (7%)	7 (7%)	7 (7%)	6 (7%)	5 (6%)	5 (6%)	5 (7%)
missing	*5 (5%)*	*11 (11%)*	*12 (13%)*	*9 (10%)*	*7 (8%)*	*6 (8%)*	*6 (8%)*
**Work absenteeism, in days**	**Parents in employment[a] n=88**	**Parents in employment[a] n=77**	**Parents in employment[a] n=74**	**Parents in employment[a] n=69**	**Parents in employment[a] n=65**	**Parents in employment[a] n=55**	**Parents in employment[a] n=56**
Sick leave, n (%)							
0	49 (56%)	46 (60%)	51 (69%)	40 (58%)	35 (54%)	30 (55%)	28 (50%)
1–5	12 (14 %)	9 (12%)	6 (8%)	7 (10%)	9 (14%)	11 (20%)	16 (29%)
6–10	2 (2%)	3 (4%)	6 (8%)	4 (6%)	4 (6%)	1 (2%)	1 (2%)
11–15	3 (3 %)	2 (3%)	2 (3%)	2 (3%)	3 (5%)	2 (4%)	1 (2%)
>15	6 (7 %)	3 (4%)	2 (3%)	3 (4%)	3 (5%)	1 (2%)	1 (2%)
missing	*16 (18 %)*	*14 (18%)*	*7 (9%)*	*13 (19%)*	*11 (17%)*	*10 (18%)*	*9 (16%)*
Vacation, n (%)							
0	43 (49%)	38 (49%)	37 (50%)	37 (54%)	33 (51%)	13 (24%)	20 (36%)
1–5	18 (20%)	16 (21%)	10 (14%)	12 (17%)	17 (26%)	15 (27%)	12 (21%)
6–10	5 (6%)	4 (5%)	11 (15%)	7 (10%)	2 (3%)	10 (18%)	8 (14%)
11–15	2 (2%)	3 (4%)	2 (3%)	1 (1%)	-	2 (4%)	3 (5%)
>15	2 (2%)	-	2 (3%)	1 (1%)	-	5 (9%)	2 (4%)
missing	*18 (20%)*	*16 (21%)*	*12 (16%)*	*11 (16%)*	*13 (20%)*	*10 (18%)*	*11 (20%)*

SD indicates standard deviation; IQR, inter quartile range; CHF, Swiss francs.

[a] Number of parents for which full-time-equivalent units were available. Some parents started to work again, explaining the increase in n at day 330.

Supplemental Table 4: Results of Little's missing completely at random test.

**Outcome variable group**	***p***
Out-of-pocket expenses	*0.82*[a]
Employment and income	*0.005*[b]
Work absenteeism	*<0.001*[b]

[a] A p-value of >0.05 suggests that there is no significant evidence to reject the null hypothesis of missing completely at random. The missing data is likely to be missing completely at random.
[b] <0.05. The missing data is likely to be missing not at random.

Supplemental Table 5: Financial support received by participating families during the 330-day care follow-up assessment.

	Day 30	Day 60	Day 90	Day 120	Day 150	Day 240	Day 330
**Financial support, in CHF**	Families n=59	Families n=54	Families n=52	Families n=50	Families n=47	Families n=42	Families n=40
Financial support, n (%)							
0	40 (68%)	31 (57%)	28 (54%)	31 (62%)	28 (60%)	21 (50%)	21 (53%)
1–499	4 (7%)	4 (7%)	5 (10%)	4 (8%)	5 (11%)	2 (5%)	1 (3%)
500–999	-	2 (4%)	3 (6%)	1 (2%)	2 (4%)	2 (5%)	1 (3%)
1’000–1’999	4 (7%)	3 (6%)	3 (6%)	4 (8%)	3 (6%)	1 (2%)	1 (3%)
2’000–2’999	5 (8%)	7 (13%)	4 (8%)	5 (10%)	5 (11%)	3 (7%)	3 (8%)
3’000–5’000	3 (5%)	1 (2%)	2 (4%)	-	1 (2%)	3 (7%)	3 (8%)
>5’000	1 (2%)	2 (4%)	5 (10%)	3 (6%)	1 (2%)	7 (17%)	10 (25%)
missing	*2 (3%)*	*4 (7%)*	*2 (4%)*	*2 (4%)*	*2 (4%)*	*3 (7%)*	-

CHF indicates Swiss francs.

Supplementary Table 6: Outcomes of the 300-day bereavement follow-up.

**Outcome**	**Day 120**<br>Parents n = 23	**Day 330**<br>Parents n = 23
Change in full-time-equivalent unit, n (%)		
No change	17 (74%)	20 (87%)
Increase	5 (17%)	1 (4%)
Decrease	1 (4%)	0 (%)
missing	-	*2 (9%)*
Change in income, n (%)		
No change	17 (74%)	20 (87%)
Increase	5 (17%)	0 (%)
Decrease	1 (4%)	1 (4%)
missing	*1 (4%)*	*2 (9%)*
Sick leave days, n (%)		
Not in employment	1 (4%)	1 (4%)
0	10 (44%)	12 (52%)
1–10	1 (4%)	1 (4%)
11–20	1 (4%)	1 (4%)
21–30	1 (4%)	1 (4%)
>30	4 (17%)	-
missing	*5 (22%)*	*7 (30%)*
Vacation days, n (%)		
Not in employment	1 (4%)	1 (4%)
0	9 (39%)	3 (13%)
1–10	5 (22%)	10 (39%)
11–20	1 (4%)	4 (17%)
21–30	-	-
>30	2 (9%)	-
missing	*5 (22%)*	*5 (22%)*

Supplemental Table 7: Complete case analyses of the effect of a child's hospital LoS on families' OOP expenses (missing completely at random) using adjusted two-way linear fixed-effects models.

**Outcome**	**Complete-case adjusted models[a]**			
	**Observations**	**Coef.**	**[95% CI]**	***p***
**Out-of-pocket expenses**				
Home healthcare supplies[b]	316	-1.47	[-3.57 to 0.63]	*0.17*
Travel and accommodation[b]	313	4.55	[2.50 to 6.60]	*<0.001*
Childcare and home help[b]	316	-3.05	[-12.00 to 5.89]	*0.50*
Special and extraordinary[b]	309	-64.09	[-182.80 to 54.01]	*0.29*

Coef. indicates coefficient; CI, confidence interval.

[a] The adjusted models are linear fixed-effects models with time and subject fixed effects adjusted for financial support and follow-up assessment period length.

[b] in Swiss francs.

Supplemental Table 8: Subgroup analysis by study participation using adjusted fixed-effects models.

Outcome	Participated for <330 days, i.e., drop-out, death of the child				Participated for the full 330 days			
	**Observations[c]**	**Coef.**	**[95% CI]**	***p***	**Observations[c]**	**Coef.**	**[95% CI]**	***p***
**Out-of-pocket expenses**								
Home healthcare supplies[a]	59	-9.19	[-26.39 to 8.00]	*0.30*	270	-1.04	[-2.99 to 0.91]	*0.30*
Travel and accommodation[a]	58	2.47	[-7.59 to 12.52]	*0.63*	270	4.04	[1.96 to 6.12]	*<0.001*
Childcare and home help[a]	59	-4.67	[-32.46 to 23.12]	*0.74*	270	-3.96	[-13.58 to 5.66]	*0.42*
Special and extraordinary[a]	59	-4.86	[-23.05 to 13.33]	*0.60*	269	-57.08	[-182.18 to 68.03]	*0.37*
**Employment and income**								
Full-time equivalent unit	96	0.00	[-0.01 to 0.01]	*0.52*	489	-0.00	[-0.00 to 0.00]	*0.98*
Income[a]	86	22.33	[-52.18 to 96.85]	*0.56*	478	2.53	[-7.43 to 12.50]	*0.62*
**Work absenteeism**								
Sick leave[b]	64	0.03	[-0.49 to 0.54]	*0.92*	337	0.03	[-0.02 to 0.09]	*0.19*
Vacation[b]	63	-0.21	[-0.53 to 0.11]	*0.22*	325	-0.06	[-0.14 to 0.02]	*0.12*

Coef. indicates coefficient; CI, confidence interval.

[a] in Swiss francs.

[b] in days.

[c] Number of observations vary because of variation in missing outcome and financial support data (see Supplemental Tables 3 and 5 for information on missing data).

Supplemental Table 9: Subgroup analysis by child hospital length of stay using adjusted fixed-effects models.

Outcome	Hospitalized for <15 days during study participation				Hospitalized for ≥15 days during study participation			
	**Observations[c]**	**Coef.**	**[95% CI]**	***p***	**Observations[c]**	**Coef.**	**[95% CI]**	***p***
**Out-of-pocket expenses**								
Home healthcare supplies[a]	191	-0.87	[-10.76 to 9.02]	*0.86*	138	-1.22	[-3.21 to 0.78]	*0.23*
Travel and accommodation[a]	190	12.62	[3.83 to 21.40]	*0.006*	138	3.21	[1.03 to 5.39]	*0.005*
Childcare and home help[a]	191	-5.92	[-48.46 to 36.62]	*0.79*	138	-5.12	[-14.00 to 3.75]	*0.26*
Special and extraordinary[a]	190	-438.68	[-1085.05 to 211.69]	*0.19*	138	6.74	[-14.36 to 27.83]	*0.53*
**Employment and income**								
Full-time equivalent unit	345	0.00	[-0.01 to 0.01]	*0.86*	240	0.00	[-0.00 to 0.00]	*0.76*
Income[a]	342	2.27	[-44.88 to 49.43]	*0.93*	222	1.18	[-6.69 to 9.04]	*0.77*
**Work absenteeism**								
Sick leave[b]	258	0.01	[-0.20 to 0.22]	*0.90*	143	0.04	[-0.03 to 0.11]	*0.23*
Vacation[b]	246	-0.14	[-0.47 to 0.19]	*0.40*	142	-0.05	[-0.13 to 0.04]	*0.27*

Coef. indicates coefficient; CI, confidence interval.

[a] in Swiss francs.

[b] in days.

[c] Number of observations vary because of variation in missing outcome and financial support data (see Supplemental Tables 3 and 5 for information on missing data).

Supplemental Table 10: Subgroup analysis by household income using adjusted fixed-effects models.

Outcome	Household income <CHF100’000				Household income ≥CHF100’000			
	Observations[c]	Coef.	[95% CI]	*p*	Observations[c]	Coef.	[95% CI]	*p*
**Out-of-pocket expenses**								
Home healthcare supplies[a]	145	-2.35	[-5.02 to 0.33]	*0.09*	176	-2.02	[-3.11 to 2.70]	*0.89*
Travel and accommodation[a]	145	6.67	[3.95 to 9.40]	*<0.001*	175	2.09	[-0.77 to 4.95]	*0.15*
Childcare and home help[a]	145	-0.97	[-2.81 to 0.88]	*0.31*	176	-6.51	[-21.46 to 8.44]	*0.40*
Special and extraordinary[a]	144	-75.39	[-232.08 to 81.31]	*0.35*	176	-38.52	[-207.79 to 130.75]	*0.66*
**Employment and income**								
Full-time equivalent unit	257	-0.00	[-0.00 to 0.00]	*0.12*	317	0.00	[-0.00 to 0.00]	*0.67*
Income[a]	258	6.49	[-1.16 to 14.14]	*0.10*	300	-2.00	[-19.57 to 15.56]	*0.82*
**Work absenteeism**								
Sick leave[b]	149	0.04	[-0.05 to 0.14]	*0.40*	250	0.03	[-0.04 to 0.09]	*0.41*
Vacation[b]	152	-0.07	[-0.19 to 0.04]	*0.18*	234	-0.05	[-0.14 to 0.05]	*0.32*

Coef. indicates coefficient; CI, confidence interval; CHF, Swiss francs.

[a] in Swiss francs.

[b] in days.

[c] Number of observations vary because of variation in missing outcome and financial support data (see Supplemental Tables 3 and 5 for information on missing data).

Supplemental Table 11: Subgroup analysis by diagnosis using adjusted fixed-effects models.

Outcome	Non-neurological				Neurological			
	**Observations[b]**	**Coef.**	**[95% CI]**	***p***	**Observations[b]**	**Coef.**	**[95% CI]**	***p***
**Out-of-pocket expenses**								
Home healthcare supplies[a]	89	-2.82	[-6.08 to 0.45]	*0.10*	240	-0.65	[-3.40 to 2.10]	*0.64*
Special and extraordinary[a]	88	-113.99	[-351.44 to 123.47]	*0.35*	240	-57.68	[-193.37 to 78.01]	*0.41*

Coef. indicates coefficient; CI, confidence interval.
[a] in Swiss francs.
[b] Number of observations vary because of variation in missing outcome and financial support data (see Supplemental Tables 3 and 5 for information on missing data).

Supplemental Table 12: Subgroup analysis by home-to-hospital travel distance using adjusted fixed-effects models.

Outcome	Home-hospital distance <21 kilometers				Home-hospital distance ≥21 kilometers			
	**Observations[b]**	**Coef.**	**[95% CI]**	***p***	**Observations[b]**	**Coef.**	**[95% CI]**	***p***
**Out-of-pocket expenses**								
Travel and accommodation[a]	120	1.66	[-0.89 to 4.20]	*0.21*	208	4.85	[2.14 to 7.55]	*<0.001*

Coef. indicates coefficient; CI, confidence interval.

[a] in Swiss francs.

[b] Number of observations vary because of variation in missing outcome and financial support data (see Supplemental Tables 3 and 5 for information on missing data).
